# Supplementary material for: Unimpaired perception of relative depth from perspective cues in strabismus
Source: R Soc Open Sci. 2020 Dec 23;7(12):200955. doi: 10.1098/rsos.200955 (PMC7813253; doi:10.1098/rsos.200955)

**Supplementary materials**

**Figure 12.** Supplementary data analysis for experiment 2.

Average difference (average rations of experiment 2 – experiment 1) for the 5 participants who took part in both experiments. Only corresponding tasks and viewing conditions (binocular) were used from the experiment 2. Error bars indicate standard deviation of the mean.

The comparison of participants’ data show that while performance in 2D C and CP staircase method tasks was similar between experiment 1 and 2, there were notable changes in participants’ 2D and 3D interval equidistance judgments in Pictorial Perspective (PP) stimuli tasks. This establish that it was the changes in testing order and more explicit explanation of the tasks that crystallised the differences between the 2D PP and 3D PP conditions in experiment 2.


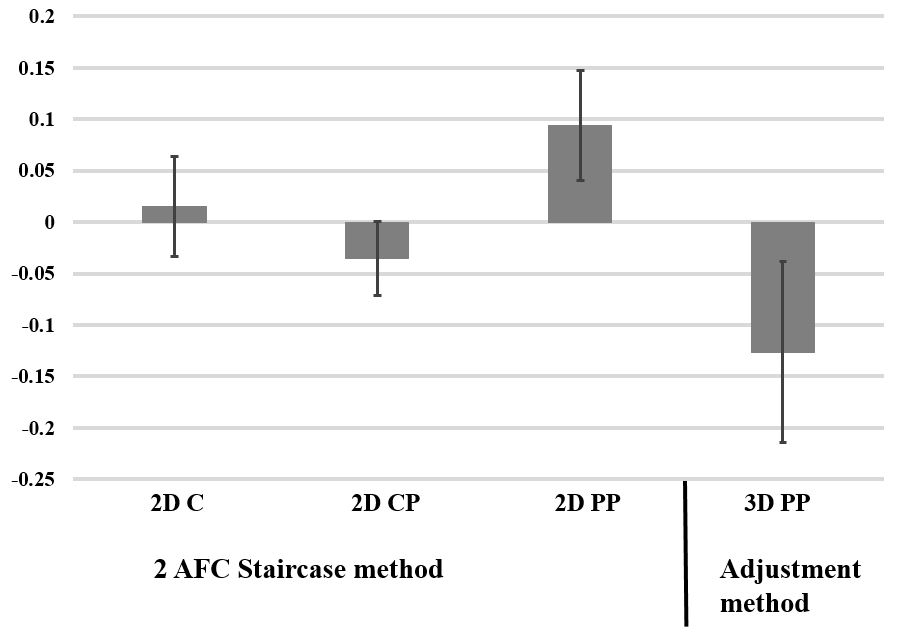

Supplement: Supplementary materials [file rsos200955supp1.docx]
